# Development and Evaluation of an Immunochromatographic Strip and a Magnetic Chemiluminescence Immunoassay for Detection of *Porcine Circovirus Type 2* Antigen

**DOI:** 10.3390/vetsci12010040

**Published:** 2025-01-09

**Authors:** Sirui Tao, Yu Duan, Yinhe Zha, Xiaxia Tong, Yulong He, Huapeng Feng, Jianhong Shu

**Affiliations:** 1 College of Life Sciences and Medicine, Zhejiang Sci-Tech University, Hangzhou 310018, China; taosirui2019@163.com (S.T.); 13625267597@163.com (Y.D.); zhayinhe2006@163.com (Y.Z.); heyl79@zstu.edu.cn (Y.H.); fenghuapeng@zstu.edu.cn (H.F.); 2 Zhejiang Hom-Sun Biosciences Co., Ltd., Shaoxing 312000, China; hzhssw@163.com; 3 Research Center of Animal Vaccines and Diagnostic Reagents, Zhejiang Sci-Tech University Shaoxing Academy of Biomedicine, Shaoxing 312090, China

**Keywords:** prokaryotic expression, immunochromatographic strip, magnetic chemiluminescence immunoassay, *porcine circovirus 2*

## Abstract

*Porcine circovirus type 2* (PCV2) is the main and primary causative agent of Postweaning Multisystemic Wasting Syndrome (PMWS). To date, immunoperoxidase monolayer assay (IPMA), indirect immunofluorescent assay (IFA), and enzyme linked immunosorbent assay (ELISA) are the most commonly diagnostic methods for detecting PCV2 antigens. However, these methods require specialized equipment and technical expertise and are suitable for laboratory use only. This study aims to develop an immunochromatographic strip and a magnetic chemiluminescence immunoassay for the detection of PCV2 antigens. The recombinant protein was constructed using a prokaryotic expression system, and the polyclonal antibody was obtained by animal experiments. Polystyrene microspheres are used as solid phase carriers to covalently bind to the amino groups of proteins to form immunoprobes. Monodisperse beads are covalently bound to antigens or antibodies as solid phases to bind antibodies or antigens in the liquid phase in a superior manner, thereby capturing and separating antigens and antibodies in the liquid phase. The immunochromatographic strip is qualitative detection method, this method can detect PCV2a strain, PCV2b strain, and PCV2d strain. The immunochromatographic strip had minimum detection limits of 10^2.89^TCID_50_/0.1 mL, 10^3.19^TCID_50_/0.1 mL, and 10^3.49^TCID_50_/0.1 mL for PCV2a/LG, PCV2b/SH, and PCV2d/JH. The results of testing PEDV (CV777 strain), PRV (HB2000 strain), CSFV (WH-09 strain), PRRS (JXA1-R strain), PPV (WH-1 strain), and ASFV (SD strain) were negative. The agreement between the immunochromatographic strip and the ELISA kit was 93.33% (140/150) and the Kappa was 0.866 (Kappa > 0.81). On the premise of ensuring sensitivity, the most important feature of the immunochromatographic strip is that this method can save time when testing; results can be obtained within 5 to 10 min. Magnetic chemiluminescence immunoassay is quantitative detection method; this method can detect PCV2 Cap proteins in swine serum, the linear range of this method was 0.25 ng/mL to 32 ng/mL and R^2^ of the standard curve was 0.9993. The limit of detection (LOD) is 0.051 ng/mL and the limit of quantitation (LOQ) is 0.068 ng/mL. The agreement between the magnetic chemiluminescence immunoassay and the ELISA kit (test PCV2 Cap proteins) was 97.14% (68/70). This method took less than 30 min to achieve results, which is less than the ELISA kit. The results of this study show that immunochromatographic strip and magnetic chemiluminescence immunoassay for PCV2 antigens had great sensitivity and specificity, which lays the foundation for PCV2 clinical detection.

## Simple Summary

This article describes the development and testing of PCV2 (*Porcine circovirus type 2*) diagnostic tools, including the expression and purification of recombinant proteins, the production of antibodies, and the validation of immunoassays. The team successfully expressed the Cap protein of PCV2 in Escherichia coli and obtained a high-purity protein by affinity chromatography purification. Subsequently, the protein was used to immunize rabbits, and polyclonal antibodies were successfully obtained and purified. The study also developed an immunochromatographic test strip and tested its sensitivity and specificity to different PCV2 strains. The results showed that the test strip showed high sensitivity to PCV and good specificity. In addition, this article also compared the immunochromatographic test strip with the traditional ELISA detection method, and the results showed that the two were highly consistent (Kappa coefficient was 0.866). Finally, the study also verified the linear range and detection limit of the magnetic chemiluminescence immunoassay, further improving the accuracy and sensitivity of PCV2 detection.

## 1. Introduction

*Porcine circovirus type 2* (PCV2) is considered to be the smallest single stranded circular DNA virus known to directly infect mammals [1,2,3]. The genome consists of 1766 nucleotides and 11 specific open reading frames (ORF) [4]. The main specific ORF is ORF1 and ORF2 [1,5,6]. ORF1 encodes two replication-related proteins (Rep and Rep’) and the capsid protein (Cap) encoded by ORF2 is the only structural protein of PCV2 [7]. At present, the Cap protein is considered to be the most immunogenic main protein of PCV2, with a molecular weight of about 27.8 kDa, consisting of 233–234 amino acids, and is closely related to PCV2 infection [8].

PCV2 has caused great harm to the pig industry. With the deepening of research [9,10,11], it was found that PCV2 not only caused Postweaning Multisystemic Wasting Syndrome (PMWS), but also caused porcine dermatitis and nephropathy syndrome (PDNS), porcine respiratory syndrome (PRDC), reproductive failure (RF), and congenital tremors (CT) [12,13,14].

PCV3 was first discovered in pig herds in North America and Asia in 2016, and later confirmed in European countries. Over time, it spread to South America and Africa. The virus can be isolated from pig serum, lymph nodes, lungs, oral fluids, and nasal swabs. In China, it has been confirmed that PCV3 genetic material exists in the serum of aborted fetuses, semen, and pigs with symptoms of reproductive disorders [15,16,17,18].

Although PCV4 was discovered in Hunan Province, China in 2019, retrospective studies have shown that PCV4 DNA has been detected in pig samples since 2012, indicating that PCV4 has been circulating in pigs for at least ten years. Since 2019, PCV4 has been reported in domestic pigs in multiple provinces of China, including Hunan, Henan, Jiangsu, Anhui, Shanxi, Guangxi, and in Inner Mongolia, as well as in South Korea. PCV4 DNA was not detected in pig samples (serum and tissue) from Europe (Italy and Spain), indicating that PCV4 may have limited geographical distribution. Therefore, further research is needed on the distribution of PCV4 in other geographical regions [19,20,21,22].

To date, polymerase chain reaction (PCR), indirect fluorescence assay (IFA), immunoperoxidase monolayer assay (IPMA), in situ hybridization (ISH), immunohistochemistry (IHC), and enzyme-linked immunosorbent assay (ELISA) had been widely used to detect PCV2 antibodies and antigens [23,24,25]. These methods should only be used in laboratories which are equipped with specialized equipment [26,27,28,29]. Therefore, a simple, rapid, and sensitive detection method is needed for antigen detection.

**Figure 1:**
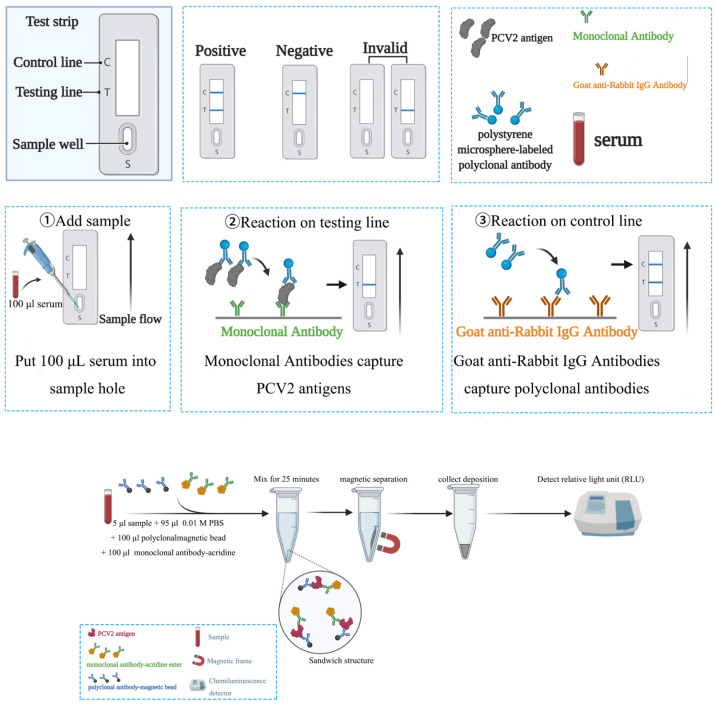
Processes of two detection methods.

Compared with traditional testing, the immunochromatographic strip and magnetic chemiluminescence immunoassay have several advantages, such as being simple, rapid, and low-cost, with no need for professional equipment [30]. The immunochromatographic strip combines polystyrene microspheres and immunochromatography. Carboxyl can be attached to the polystyrene microspheres, and polystyrene microspheres can be linked to the aminos of antigens or antibodies by carboxyl [30,31,32,33]. The monoclonal antibodies were fixed at the test line and goat anti-rabbit IgG secondary antibodies were fixed at the control line on NC membrane [32,34]. The appearance of color depends on the antigen–antibody reaction. Polyclonal antibody-labeled polystyrene microspheres can catch antigens in a sample, monoclonal antibodies, which are in test line, can also catch antigens; therefore, these components compose a sandwich structure—monoclonal antibodies link the nitrocellulose membrane (NC membrane) by hydrophobic interaction. Finally, this sandwich structure stays at test line and a blue-colored band will appear at the test line. The goat anti-rabbit IgG secondary antibodies which were fixed at the control line can catch polyclonal antibody-labeled polystyrene microspheres which are redundant, and a blue-colored band will appear at the control line. The sample is positive when two blue lines appear on the strips. The sample is negative when one blue line is at the control line. No blue line and one blue at the test line show that the result is invalid. Figure 1 shows the fabrication process of both methods.

Now, raw material suppliers can provide polychrome polystyrene microspheres; the red polystyrene microsphere and bule polystyrene microsphere are widely used in immunochromatographic strips. The appearance of color depends on the antigen–antibody reaction; so, we do not need to add any substrates or enzymes to the strip. Instead of polystyrene microspheres, many other alternatives can be chosen to develop strip, such as colloidal gold or fluorescent microspheres. Their sensitivity and specificity will change when we change label because these labels have different physical and chemical properties.

A magnetic chemiluminescence immunoassay combines magnetic particles and chemiluminescent immunoassay (CLIA). Briefly, the double antibody sandwich exists when antibody-magnetic particles and an antibody-acridine ester bind to a PCV2 antigen. After magnetic particle separation, acridine esters which were on monoclonal antibodies can generate an optical signal by buffer A and B; the detection machine will record the relative light unit (RLU). RLU is proportional to the PCV2 antigen [35].

The cores of magnetic particles are iron oxides; magnetic particles have super-paramagnetic effect. These magnetic particles can quickly gather when there is an external magnetic field. Meanwhile, magnetic particles can be separated quickly when the external magnetic field is removed [36,37,38]. Magnetic particles also have a larger specific surface area; magnetic particles provide a more adequate reaction environment for immune response. Carboxyl can be attached to the surface of magnetic particles, and magnetic particles can be linked to the aminos of antigen or antibody by carboxyl [39,40,41].

The acridine ester compound is the core of the magnetic chemiluminescence immunoassay (CLIA). Compared with other chemiluminescence systems, acridine ester compounds have low luminescence interference and low background and do not need enzymes or catalysts [42,43,44]. They are suitable for all-automatic and kinetic detection. In addition, this type of compound also has very good stability; many advantages have resulted in it gradually occupying the main position in actual analysis and detection applications [45,46,47]. In the future, the acridine ester compound will become the chemiluminescent substance with the greatest research and application value [48,49,50].

Currently, the ELISA kit is the most common method for detecting *porcine circovirus type 2* infection, but it takes several hours to obtain results. Our team hope to develop one or two methods to detect *porcine circovirus type 2* infection within 30 min. So, we chose the immunochromatographic strip and the magnetic chemiluminescence immunoassay. Immunochromatographic strip is a qualitative analysis method. Its greatest feature is that the results can be obtained in 15 min. Meanwhile, magnetic chemiluminescence immunoassay takes less than 30 min; moreover, we can accurately know the number of antigens in serum by using magnetic chemiluminescence immunoassay.

In this study, immunochromatographic strip and magnetic chemiluminescence immunoassay were successfully developed and applied to test for PCV2 antigen. Immunochromatographic strip can achieve accurate results within 10 min and magnetic chemiluminescence immunoassay can achieve accurate results within 25 min. In summary, two methods were used to improve the speed and simplicity of PCV2 diagnosis without compromising sensitivity and specificity. Immunochromatographic strip and magnetic chemiluminescence immunoassay provide new ideas for the detection of PCV2.

## 2. Materials and Methods

### 2.1. Clinical Samples, Cells, Viruses, and Enzyme

One hundred and fifty serum specimens from PCV2-suspected pigs were preserved in the College of Life Sciences and Medicine, Zhejiang Sci-Tech University, Hangzhou, China. All serum specimens were stored at −80 °C.

PK15 cells (ATCC^®^ CCL-33), Dulbecco’s modified eagle medium (DMEM, Gibco, Waltham, MA, USA), fetal bovine serum (FBS, Gibco, MA, USA).

XohI restriction enzyme (TKARA, Tokyo, Japan), NcoI restriction enzyme (TKARA, Tokyo, Japan).

Urea (National pharmaceutical reagent, Shanghai, China), Bovine Serum Albumin (BSA, Solarbio, Hangzhou, China), Ni-NTA (Solarbio, Hangzhou, China), SDS gel electrophoresis (Solarbio, Hangzhou, China), BCA assay (Solarbio, Hangzhou, China), Protein A kit (Solarbio, Hangzhou, China), EDC (RHAWN, Hangzhou, China), polystyrene microsphere (Huge Biotechnology, Shanghai, China), magnetic beads (Huge Biotechnology, Shanghai, China), Matrix SephadexTM G-25 resin (Solarbio, Hangzhou, China).

Goat anti-Rabbit IgG (H+L) Highly Cross-Adsorbed Secondary Antibody was supplied by Thermo Fisher Scientific. Mouse monoclonal antibody against PCV2-Cap was supplied by Hangzhou DaYao Biotechnology Co., Ltd. (Hangzhou, 310018, China).

Strain origins genotypes and accession numbers are shown in Table 1.

### 2.2. Antibodies and Microsphere

Goat anti-Rabbit IgG (H+L) Highly Cross-Adsorbed Secondary Antibody was supplied by Thermo Fisher Scientific. Mouse monoclonal antibody against PCV2-Cap was supplied by Hangzhou DaYao Biotechnology Co., Ltd. Polystyrene microsphere and magnetic microspheres were produced by Shanghai Huge Biotechnology Co., Ltd. (Shanghai, 200000, China).

### 2.3. Preparation of Recombinant Cap Protein of PCV2

In this study, the PCV2 ORF2 (GenBank DQ231512.1) complete coding DNA sequences (CDS) and primer sequences were both synthesized by GENEWIZ Biotechnology Co., Ltd. (Suzhou, 215000, China). The forward primer sequence is 5′-GCCCATGGGCAAAAATGGCATCTTCAAC-3′ and the reverse primer is 5′-GCCTCGAGAGGGTTAAGTGGGGGGTCTT-3′.

XohI and NcoI were used to digest the PCR product, then the purified PCR product was cloned into the pET-28a vector. DNA sequencing was used to confirm the construct (GENEWIZ, Suzhou, China). Finally, recombinant plasmids were transformed into *E. coli* BL21-competent cells.

An optical density of 0.8 at 600 nm was measured using a positive clone of recombinant protein grown at 37 °C in lysogeny broth supplemented with 50 μg/mL kanamycin. In the next step, 0.5 mM of IPTG was added to lysogeny broth. Approximately 15 h after induction at 30 °C, cells were harvested by centrifugation (12,000 rpm for 15 min) and resuspended in 10 mL of 10 mM PBS (pH 7.2). The ice-cold cells were broken down by sonication and centrifuged at 12,000 rpm for 25 min. The precipitation was collected; the protein was purified using Ni-NTA resin as specified by the manufacturer.

The protein solution was added into a pre-treated dialysis bag. Firstly, buffer I (8 mol/L urea, 0.01 M PBS based solution) was used to dialysis protein solution.

Then, buffer II (6 mol/L urea, 0.01 M PBS based solution), buffer III (4.5 mol/L urea, 0.01 M PBS based solution), buffer IV (3.5 mol/L urea, 0.01 M PBS based solution), buffer V (2.5 mol/L urea, 0.01 M PBS based solution), buffer VI (1.5 mol/L urea, 0.01 M PBS based solution), and buffer VII (0.5 mol/L urea, 0.01 M PBS based solution) were used to perform dialysis of the protein solution in turn; the liquid changing interval was 12 h.

Finally, 0.01 M PBS was used to perform dialysis of the protein solution.

High concentrations of urea (8 mol/L) can denature PCV2 Cap protein completely; we reduced the concentration of urea gradually so that PCV2 Cap protein will be refolded gradually.

Before storing at −80 °C, the protein was tested by SDS PAGE.

### 2.4. Preparation of Polyclonal Antibody

Two healthy female rabbits (6 to 8 weeks of age) were provided by Zhejiang University for the purposes of this study. Freund’s complete adjuvant (FCA) was used to emulsify 100 μL Cap solution (1 μg/μL) before injecting it into the rabbit on day 0. For secondary and booster injections, Freund’s incomplete adjuvant (FIA) was used instead of FCA to prevent inflammation and lesions due to the formation of granulomas on day 14 and day 28. Vaccination of the last immunization was performed without adjutant on day 42. At the same time, a PBS negative control group was set up. An enzyme-linked immunosorbent assay kit (ELISA kit) was used to analyze the antibody level.

According to aseptic purification strategies, specific polyclonal antibodies can be isolated from serum using a Protein A kit. The polyclonal antibody also be tested using sodium dodecyl sulfate-polyacrylamide gel electrophoresis and the BCA protein determination method.

The ethics committee of Zhejiang Sci-Tech University’s College of Life Sciences has reviewed and approved the study on mice, and this study was conducted according to its guidelines (Code: 20180401, Date: 2 April 2018).

### 2.5. Conjugation of Polyclonal Antibody with Polystyrene Microsphere

A measure of 0.9 mL of 0.02 M sodium borate buffer was incubated with 0.1 mL of polystyrene microsphere solution with rapid stirring (220 rpm) for 5 min; then, 0.2 mL 1-(3-Dimethylaminopropyl)-3-ethylcarbodiimide hydrochloride solution (EDC, 1 mg/mL) and 0.1 mL of polyclonal antibody was added to the stirred solution. After the mixture was stirred for 15 min at 25 °C, 1 mL of BSA solution (2 mg/mL) was added to the stirred solution and mixture was continuously stirred for 60 min. Next, the stirred solution was harvested by centrifugation (12,000 rpm for 15 min) and the precipitation is the polystyrene microsphere-labeled polyclonal antibody. As a final step, the precipitation was added to 1 mL of 0.02 M sodium borate buffer (containing 0.1% Proclin300, 1% BSA); then, the solution was stored at 4 °C. This solution can be used as the immunoprobe.

Before the assembly of the test strips, 500 μL of immunoprobe is dropped onto the front of the sample pad. The treated sample pad was placed in an oven to dry.

### 2.6. Immobilization of Test Line and Control Line

Monoclonal antibody solution (2 mg/mL) and goat anti-Rabbit IgG (2 mg/mL) were injected into pressure pumps in platform-sensing dispenser. Two lines (testing line and control line) were draw on the nitrocellulose (NC) membrane, and there was approximately 0.5 cm between the 2 lines; NC membranes must be stored in the oven for 6 h [32].

### 2.7. Assembly of Immunochromatographic Strip

**Figure 2:**
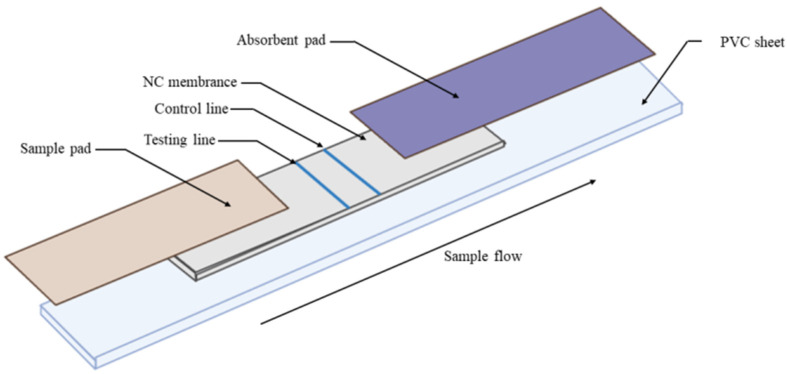
Structure schematic of test trip.

Each strip contained a sample pad, NC membrane, and absorbent pad (Figure 2). An adhesive plastic backing had the NC membrane pasted in the center. Overlapping 1 mm on the top and bottom of the NC membrane, the absorbent pad and sample pad were then pasted together. Finally, the boards were cut into 3.0 mm wide strips [51].

### 2.8. Evaluation of Immunochromatographic Strip

#### 2.8.1. Preparation of Sample

An immunochromatographic strip can be used to detect PCV2 strain in cell culture media (PK-15 cell) or swine serum. Tissue culture infectious dose (TCID_50_) assay was used to detect the virus titer of different genotype PCV2 strains. Add 100 μL cell culture media to the sample pad and wait 5–10 min to observe the result. After centrifugation, swine serum was obtained from whole blood. Add 100 μL swine serum to the sample pad and wait 5–10 min to observe the result.

#### 2.8.2. Sensitivity Test of Immunochromatographic Strip

We collected 3 strains. They are PCV2a/LG (10^5^ TCID_50_/0.1 mL), PCV2b/SH (10^5^ TCID_50_/0.1 mL), and PCV2d/JH (10^5^ TCID_50_/0.1 mL). Three PCV2 strains were used to evaluate the sensitivity of the immunochromatographic strip. Three viruses were serially diluted tenfold from 10^5^ TCID_50_/0.1 mL to 10^2.29^ TCID_50_/0.1 mL. A PK-15 cell culture was used for blank control. Immunochromatographic strips were used to test these virus diluent solutions; each diluent solution should be tested 3 times.

#### 2.8.3. Evaluation of the Detection Range of Immunochromatographic Strip

We collected 5 PCV2 strains. They are PCV2a/LG (10^5^ TCID_50_/0.1 mL), PCV2a/SZ (10^5^ TCID_50_/0.1 mL), PCV2b/SH (10^5^ TCID_50_/0.1 mL), PCV2d/JH (10^5^ TCID_50_/0.1 mL), and PCV2d/WZ (10^5^ TCID_50_/0.1 mL). These different PCV2 genotype strains were used to evaluate the detection range of the immunochromatographic strip.

#### 2.8.4. Specificity Test of Immunochromatographic Strip

We prepared 20 PCV2-positive serum samples, 10 PCV2-negative serum samples, 5 PEDV-positive serum samples, 5 PRV-positive serum samples, 5 CSFV-positive serum samples, 5 PRRS-positive serum samples, 5 PPV-positive serum samples, ASFV PCV1-positive serum samples, and 1 PCV1 strain. We used immunochromatographic strips to test serum samples and strain; each serum sample or strain was tested 3 times.

We prepared cell culture mediums of PEDV (CV777 strain), PRV (HB2000 strain), CSFV (WH-09 strain), PRRS (JXA1-R strain), PPV (WH-1 strain), and ASFV (SD strain). A measure of 100 μL cell culture medium was added onto the sample pad; each sample should be tested 3 times.

### 2.9. Immunochromatographic Strip Compared with ELISA 

In total, 150 serum samples were collected from three swine herds in Zhejiang province and the immunochromatographic strip and Porcinecircovirus 2 Cap ELISA kit (antigen testing, Beijing Median Biotek Co., Ltd., (Beijing, 100000, China) were used to test the 150 serum samples.

The immunochromatographic strip is qualitative detection method, Porcinecircovirus 2 Cap ELISA kit is quantitative detection method. Therefore, we used a Kappa test to analyze the differences between the two sets of data.

The Kappa consistency test is a statistical method used to evaluate the consistency level of two or more evaluators on categorical data. It not only tells us whether there is consistency between evaluators, but also quantifies the degree of consistency through a Kappa value.

Kappa values between 0.61 and 0.80 were considered to indicate ‘high’ consistency, while those between 0.81 and 1.00 indicated ‘extremely strong’ consistency.

### 2.10. Development of Magnetic Chemiluminescence Immunoassay

#### 2.10.1. Conjugation of Polyclonal Antibody with Magnetic Bead

A measure of 0.9 mL of 0.02 M sodium borate buffer was incubated with 0.1 mL of magnetic bead solution with rapid stirring (220 rpm) for 5 min; then, 0.2 mL 1-(3-Dimethylaminopropyl)-3-ethylcarbodiimide hydrochloride solution (EDC, 1 mg/mL) and 0.1 mL of anti-PCV2-Cap polyclonal antibody (1 mg/mL) was added to the stirred solution. After the mixture was stirred for 15 min at 25 °C, 1 mL of BSA solution (2 mg/mL) was added to the stirred solution and the mixture was continuously stirred for 60 min. Next, the stirred solution was harvested by centrifugation (12,000 rpm for 15 min) and the precipitation was the magnetic beads-labeled polyclonal antibody. As a final step, the precipitation was added to 1 mL of 0.02 M sodium borate buffer; then, the solution was stored at 4 °C.

#### 2.10.2. Conjugation of Monoclonal Antibody with Acridine Ester

Measures of 0.1 mL of monoclonal antibody (1 mg/mL) and 7.87 μL acridine ester solution (0.5 mg/mL) were added to 0.8 mL of 0.02 M sodium borate buffer; the mixture was stirred (220 rpm) for 3 h. Then, conjugation was ended by the addition of 10 μL lysine solution (10% lysine). Finally, the stirred solution was purified using a Matrix Sephadex™ G-25 Fine.

### 2.11. Evaluation of Magnetic Chemiluminescence Immunoassay

#### 2.11.1. Preparation of Sample Solution

In the testing process of magnetic chemiluminescence immunoassay, place 5 μL of serum sample and 95 μL of sample diluent (0.01 M PBS) into a reaction tube and mix them together. Place 100 μL of probe solution and 100 μL of acridine ester-labeled antibody into a reaction tube and mix them together. The reaction tube should be stored at 37 °C for 25 min. Place the reaction tube on a magnetic rack for magnetic separation; the reaction solution should be discarded and 1000 μL of washing buffer should be added into tube for cleaning. Put the reaction tube into the automatic chemistry system; the system will inject 100 μL buffer A and 100 μL buffer B into the tube. Finally, the system will detect the relative light unit (RLU).

#### 2.11.2. Evaluation of the Linear Range of the Magnetic Chemiluminescence Immunoassay

In this study, we prepared PCV2 Cap solutions of different concentrations (0.25 ng/mL, 0.5 ng/mL, 1 ng/mL, 2 ng/mL, 4 ng/mL, 8 ng/mL, 16 ng/mL, 32 ng/mL). Each sample corresponds to one relative light unit (RLU). Then, the standard curve for detecting PCV2 antigen was developed; meanwhile, the coefficients of determination (R^2^) of standard curve are also recorded to show the linear relationship (R^2^ > 0.9).

#### 2.11.3. LOD and LQD of Magnetic Chemiluminescence Immunoassay

According to the Clinical and Laboratory Standards Institute (NCCLS) guidelines, one negative sample and one low-concentration sample were selected to manufacture a standard curve. The negative sample was tested 20 times and, calculating average (Mean) and standard deviation (SD), Mean + 2SD and Mean + 10SD were substituted into the standard curve; the result of Mean + 2SD was LOD and result of Mean + 10SD was LOQ.

#### 2.11.4. Specificity Test of Magnetic Chemiluminescence Immunoassay

We prepared 5 PCV2-positive serum samples, 5 PCV2-negative serum samples, 5 PRV-positive serum samples, 5 PRRSV-positive serum samples, 5 PEDV-positive serum samples, and 5 CSFV-positive serum samples; each serum sample was tested 3 times.

### 2.12. Magnetic Chemiluminescence Immunoassay Compared with ELISA

Magnetic chemiluminescence immunoassay and the Porcinecircovirus 2 Cap ELISA kit (antigen testing, Beijing Median Biotek Co., Ltd.) were used to test 70 serum samples. A Bland–Altman diagram was used to analyze the results between the magnetic chemiluminescence immunoassay and Porcinecircovirus 2 Cap ELISA kit. The most important reason for choosing a Bland–Altman diagram is that the magnetic chemiluminescence immunoassay and Porcinecircovirus 2 Cap ELISA kit both are quantitative analysis methods. We hope to analyze the difference between them for the same serum.

The Bland–Altman diagram is a visual display method for consistency measurement. After the relevant calculation of the measured data, the scatter points are displayed. If the scatter points are within the confidence interval (generally within the range of 1.96 standard deviations of the difference), then the data have a good consistency level.

## 3. Results

### 3.1. Expression and Purification of the Cap Protein in E. coli and Western Blot Identification

**Figure 3:**
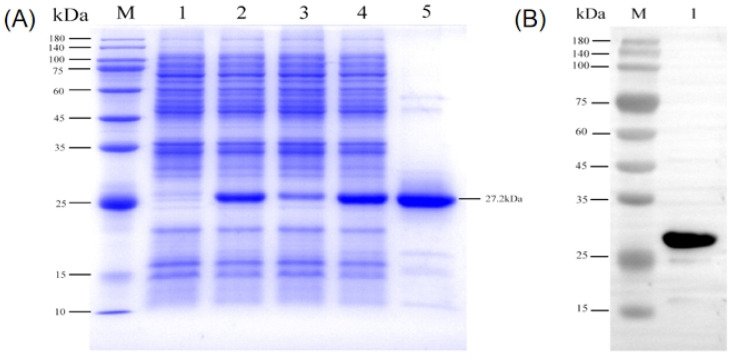
Purification of the Cap protein and western blot. (**A**) Identification of purified recombinant proteins by SDS-PAGE: M—Protein molecular weight standard; 1—Not induced; 2—After induction; 3—Upper clear; 4—Precipitation; 5—Purified recombinant Cap protein. (**B**) Western blot analysis of purified recombinant Cap protein: M—Protein molecular weight standard; 1—Purified recombinant Cap protein.

The recombinant Cap protein was expressed and purified from *E. coli* (BL21). An inserted 6 × histidine tag is attached to this approximately 27.2 kDa protein. After sonication treatment, SDS-PAGE detection showed that the expressed target protein was presented mainly in the form of inclusion body. BCA protein assay was used to test the concentration of the Cap protein purified by affinity chromatography after renaturation. Purification resulted in protein with over 90% purity according to SDS-PAGE (Figure 3).

### 3.2. Purification of Polyclonal Antibody

**Figure 4:**
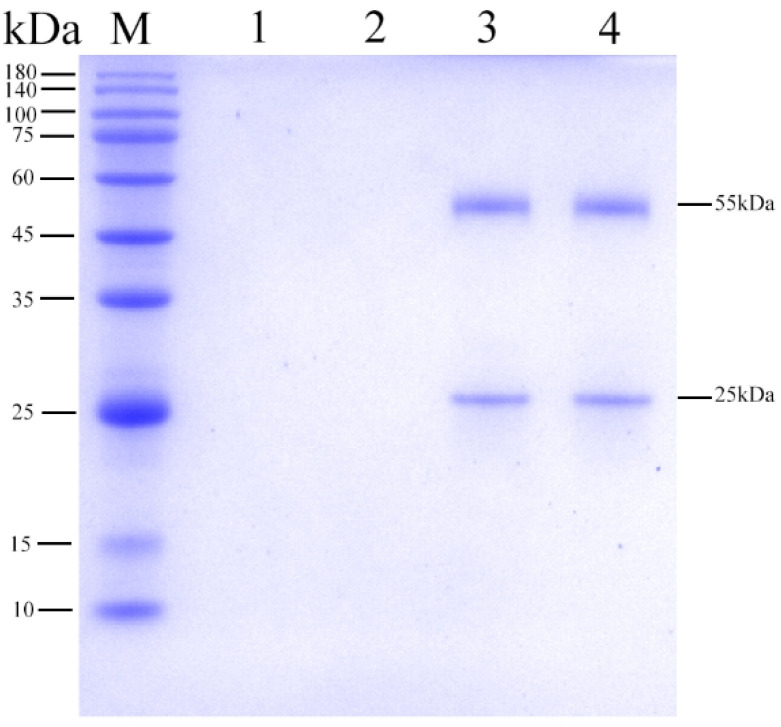
Purification of the polyclonal antibodies. M—maker; 1, 2—unimmunized serum; 3, 4—immunized serum.

After four immunizations, polyclonal antibodies were produced and existed in the serum of the rabbits. Blood was collected from the heart and serum was obtained by centrifuge (3000 rpm). Polyclonal antibodies were purified using saturated ammonium sulfate. Purification resulted in polyclonal antibody according to SDS-PAGE (Figure 4). Polyclonal antibodies were composed of two chains; light chain is 25 kDa and heavy chain is 55 kDa.

### 3.3. Sensitivity of Immunochromatographic Strip

**Figure 5:**
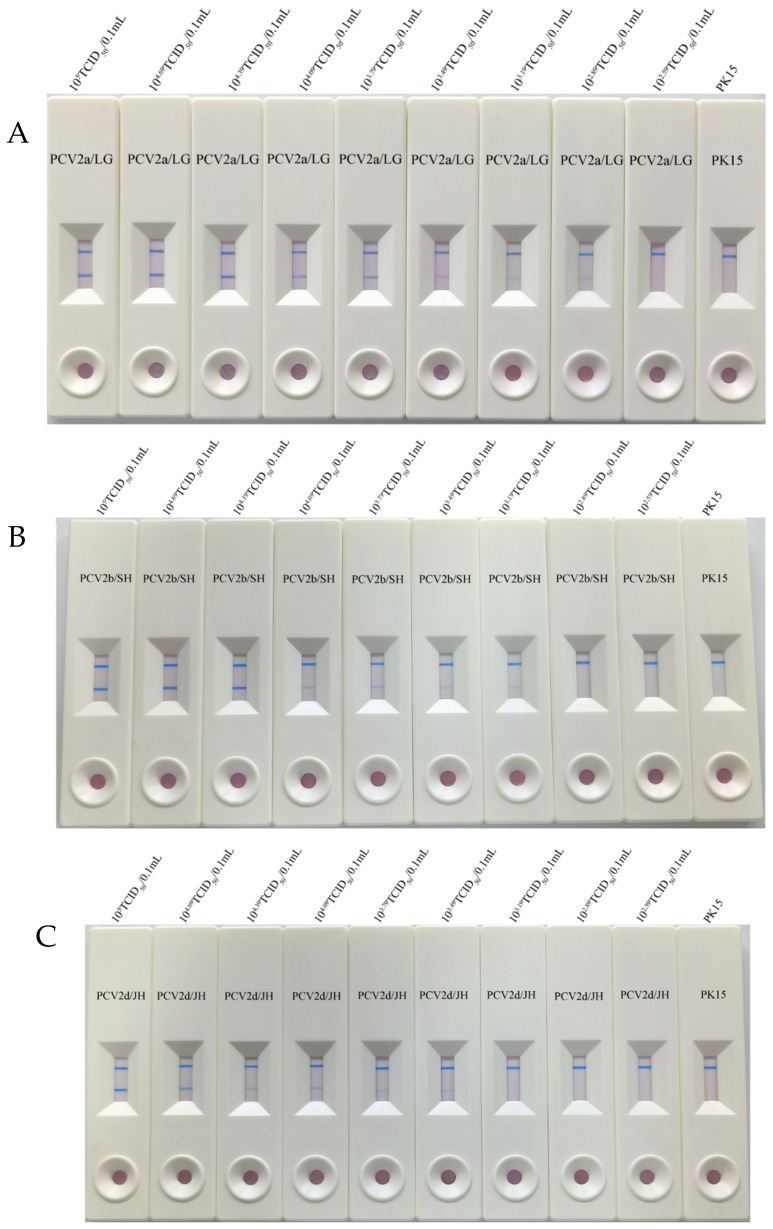
Sensitivity and specificity of the test strip. (**A**) PCV2a/LG; (**B**) PCV2b/SH; (**C**) PCV2d/JH.

In this study, PCV2a/LG (10^5^ TCID_50_/0.1 mL), PCV2b/SH (10^5^ TCID_50_/0.1 mL) and mL, 10^3.19^ TCID_50_/0.1 mL, 10^3.49^ TCID_50_/0.1 mL (Figure 5A–C).

We can draw a conclusion that the immunochromatographic strip was more sensitive to PCV2a strains and PCV2b strains than PCV2d strains.

### 3.4. Detection Range of Immunochromatographic Strip

**Figure 6:**
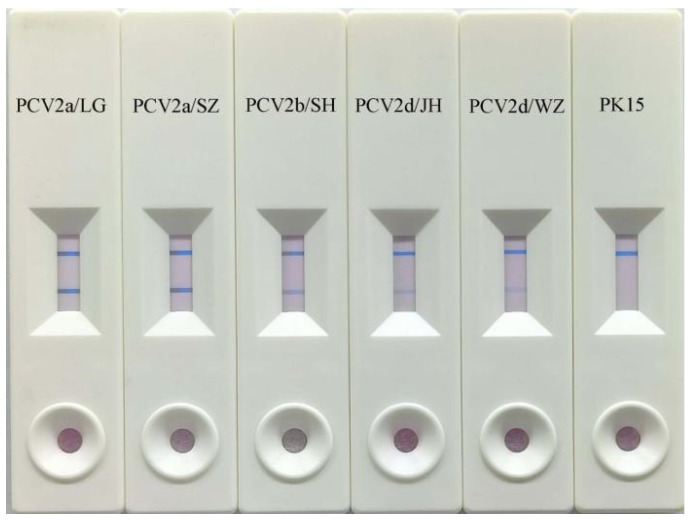
Detection range of immunochromatographic strips: PCV2a/LG (10^5^ TCID_50_/0.1 mL), PCV2a/SZ (10^5^ TCID_50_/0.1 mL), PCV2b/SH (10^5^ TCID_50_/0.1 mL), PCV2d/JH (10^5^ TCID_50_/0.1 mL), and PCV2d/WZ (10^5^ TCID_50_/0.1 mL). PK-15 cell culture is the blank control.

We collected five PCV2 strains. They are PCV2a/LG (10^5^ TCID_50_/0.1 mL), PCV2a/SZ (10^5^ TCID_50_/0.1 mL), PCV2b/SH (10^5^ TCID_50_/0.1 mL), PCV2d/JH (10^5^ TCID_50_/0.1 mL), and PCV2d/WZ (10^5^ TCID_50_/0.1 mL). The results of all experimental groups were positive. We can reach a conclusion that immunochromatographic strips can be used to test PCV2a, PCV2b, and PCV2d (Figure 6).

### 3.5. Specificity of Immunochromatographic Strip

**Figure 7:**
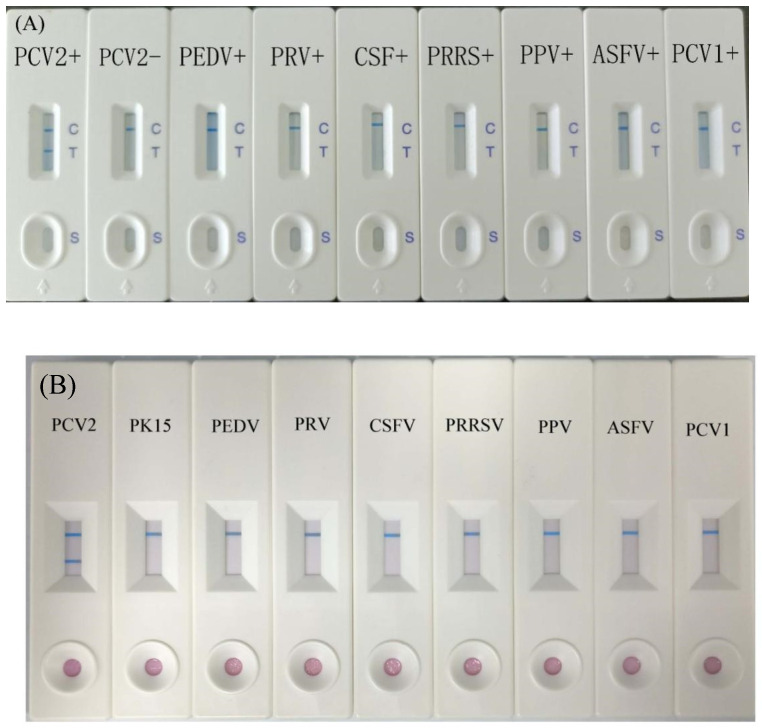
Sensitivity and specificity of the test strip. (**A**) The results of serum sample. (**B**) The results of cell culture medium.

PCV2-positive serum samples, PCV2-negative serum samples, PEDV-positive serum samples, PRV-positive serum samples, CSFV-positive serum samples, PRRS-positive serum samples, PPV-positive serum samples, and an ASFV-positive serum sample, and PCV1 strain solution were tested using the immunochromatographic strip; the results showed that positive results only appeared in PCV2-positive serum samples. Other experimental groups showed negative results (Figure 7A).

Moreover, we collect cell culture medium of PEDV (CV777 strain), PRV (HB2000 strain), CSFV (WH-09 strain), PRRS (JXA1-R strain), PPV (WH-1 strain), ASFV (SD strain), and PCV1 (D strain). All results were negative (Figure 7B).

Based on the results, immunochromatographic strips can test serum samples and strain samples.

### 3.6. Comparison of ELISA Kit with Immunochromatographic Strip

We prepared 150 serum samples and used the developed immunochromatographic strip and ELISA kit to test serum samples (Table 2).

The Kappa statistic was used to prove that the immunochromatographic strip showed a high level of consistency with the enzyme-linked immunosorbent assay (ELISA) kit. The agreement between the immunochromatographic strip and ELISA kit was 93.33% (140/150). The Kappa coefficient between immunochromatographic strip and ELISA kit was 0.866 (Kappa > 0.810); the two methods have almost perfect agreement (Table 2).

### 3.7. Linear Range of the Magnetic Chemiluminescence Immunoassay

**Figure 8:**
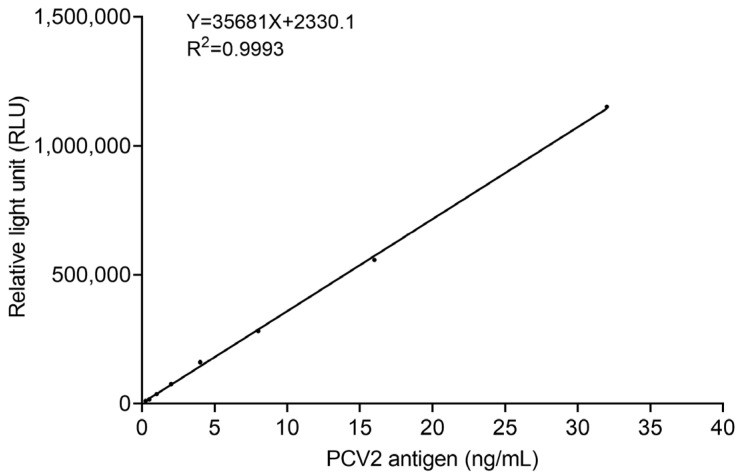
Standard curves of magnetic chemiluminescence immunoassay (0.25 ng/mL, 0.5 ng/mL, 1 ng/mL, 2 ng/mL, 4 ng/mL, 8 ng/mL, 16 ng/mL, 32 ng/mL).

PCV2 Cap solutions of different concentrations (0.25 ng/mL, 0.5 ng/mL, 1 ng/mL, 2 ng/mL, 4 ng/mL, 8 ng/mL, 16 ng/mL, 32 ng/mL) were prepared; each sample corresponds to one relative light unit (RLU) and standard curves were established using SPSS. The R^2^ of standard curves is 0.9993; there is a great correlation between PCV2 antigen and RLU when the concentration of PCV2 antigen is 0.25 ng/mL to 32 ng/mL (Figure 8).

### 3.8. The Limit of Detection (LOD) and the Limit of Quantitation (LOQ)

According to the Clinical and Laboratory Standards Institute (NCCLS) guidelines, one negative sample and one low-concentration sample were selected to manufacture a standard curve. The negative sample was tested 20 times and, calculating average (Mean) and standard deviation (SD), Mean + 2SD and Mean + 10SD were substituted into the standard curve; the result of Mean + 2SD was LOD and result of Mean + 10SD was LOQ (Table 3).

### 3.9. Specificity of Magnetic Chemiluminescence Immunoassay

In this study, we collected five PCV2-positive serums, five PCV2-negative serums, five PRV-positive serums, five PRRSV-positive serums, five PEDV-positive serums, and five CSFV-positive serums. These serums were tested by magnetic chemiluminescence immunoassay. The result show that positive results only appeared when we tested PCV2-positive serums. Meanwhile, the results of other experimental groups were negative (Table 4).

We collected cell culture mediums of PCV2a/LG (10^5^ TCID_50_/0.1 mL), PCV2a/SZ (10^5^ TCID_50_/0.1 mL), PCV2b/SH (10^5^ TCID_50_/0.1 mL), PCV2d/JH (10^5^ TCID_50_/0.1 mL), and PCV2d/WZ (10^5^ TCID_50_/0.1 mL). The PK-15 cell culture was a blank control; the results are shown (Table 5).

We tested the cell culture medium of PEDV (CV777 strain), PRV (HB2000 strain), CSFV (WH-09 strain), PRRS (JXA1-R strain), and PPV (WH-1 strain); the results are shown (Table 5).

### 3.10. Comparison of ELISA Kit and Magnetic Chemiluminescence Immunoassay

**Figure 9:**
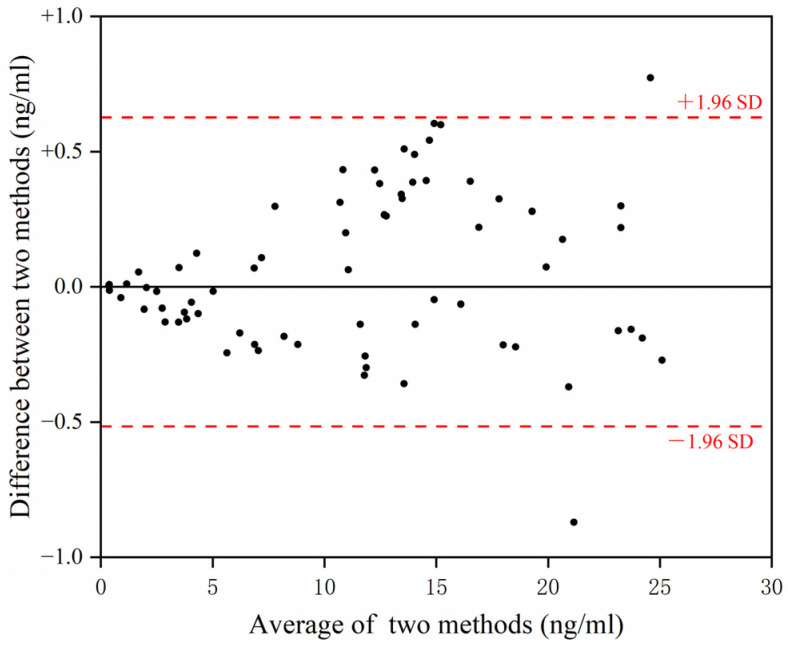
Agreement between immunochromatographic strip and ELISA kit.

In total, 70 serum samples were tested by magnetic chemiluminescence immunoassay and ELISA kit. The Bland–Altman test was used to obtain the agreement between the magnetic chemiluminescence immunoassay and ELISA kit. For the same sample, there was a high level of consistency between the immunochromatographic strip and the ELISA kit when the difference in result was between +1.96SD and −1.96SD (Figure 9). Only two samples had a substantial difference. The agreement between the magnetic chemiluminescence immunoassay and ELISA kit was 97.14% (68/70). The results showed that two measures had high consistency.

## 4. Discussion

*Porcine circovirus 2* (PCV2) is the main and primary causative agent of Postweaning Multisystemic Wasting Syndrome (PMWS), which has caused significant economic losses in the swine industry. Several solution techniques have been applied to test for *Porcine circovirus 2.* Immunoperoxidase monolayer assay (IPMA), indirect immunofluorescent assay (IFA), and enzyme-linked immunosorbent assay (ELISA) are the most common diagnostic methods for detecting PCV2 antibodies. However, these methods require specialized equipment and technical expertise and are suitable for laboratory use only.

A technology was needed that has several advantages over traditional immunoassays, such as simplicity of procedure, rapid operation, immediate results, low cost, and no requirements for technical expertise or specialized equipment. In this study, immunochromatographic (detection time 5 min) and magnetic chemiluminescence immunoassay (detection time 20 min) possess high sensitivity and specificity and may be useful for clinical laboratories and rapid diagnosis in the field. In this study, the recombinant Cap protein was expressed and SDS PAGE showed a new protein with a molecular weight of 27.8 KD. Then, the rabbits were immunized with the purified Cap protein to produce multiclonal antibodies.

A rapid (less than 10 min) immunochromatographic strip, using polystyrene microspheres, was successfully developed and applied in the detection of the porcine circovirus 2 (PCV2) antigen. The polyclonal antibody and goat anti-Rabbit IgG secondary antibody were blotted on the nitrocellulose membrane for the test line and control line. The specificity of the strip was 100% (65/65). Regarding sensitivity, the immunochromatographic strip could test for a concentration of Cap antigen as low as 0.25 μg/mL. In total, 150 clinical swine serum samples were detected both by strip and the commercial ELISA kit. The agreement of immunochromatographic strip and ELISA kit was 93.33%. It showed that this strip possesses high sensitivity and specificity and may be useful for clinical laboratories and rapid diagnosis in the field.

We created a magnetic chemiluminescence immunoassay; there are two important features. First, the polyclonal antibody is conjugated with a magnetic bead. Second, the monoclonal antibody is conjugated with an acridine ester.

The magnetic chemiluminescence immunoassay had a high correlation and consistency. This method had a good linear relationship, between 0.25 ng/mL and 25 ng/mL; the R^2^ is 0.9993. The limit of detection is 0.051 ng/mL and the limit of quantitation is 0.058 ng/mL. In the contrast experiment of 70 serum specimens, results showed that the agreement between the magnetic chemiluminescence immunoassay and ELISA kit was 97.14% (68/70). The magnetic chemiluminescence immunoassay and ELISA kit had high correlation and consistency.

In summary, we established immunochromatographic and magnetic chemiluminescence immunoassay to detect the antigen against PCV2. The assays showed good performance in terms of sensitivity and specificity and have certain application value, which could potentially be used for monitoring the immune responses in pigs to PCV2 infections and vaccination as well.

## 5. Conclusions

PCR, ELISA, and gene chip detection are the main methods for the detection of PCV2, but each has its own limitations, such as the probability of false positives in PCR detection [52], the complexity and low-sensitivity of ELISA process [5], and the high cost and difficulty of gene-chip detection [53]. This study has successfully developed a solution to this problem, developed and applied an immunochromatographic strip and magnetic chemiluminescence immunoassay to test for PCV2 antigen. The immunochromatographic strip can achieve accurate results within 10 min; magnetic chemiluminescence immunoassay can achieve accurate results within 25 min. The two methods were used to improve the speed and simplicity of PCV2 diagnosis without compromising sensitivity and specificity. The immunochromatographic strip and magnetic chemiluminescence immunoassay provide new ideas for the detection of PCV2.

## Figures and Tables

**Table 1:** Origins of the PCV2 strains used in this study.

Name	Isolate Region	Isolate Time	Genotype	Genotype	Genome Length	Accession Numbers
LG	China	2008	8839.70	PCV2a	1767 bp	HM038034
SZ	China	2016		PCV2a	1767 bp	KX814351
SH	China	2006		PCV2b	1767 bp	HM038027
JH	China	2017		PCV2d	1767 bp	MG245867
WZ	China	2016		PCV2d	1767 bp	MK604498

**Table 2:** Comparison of the developed immunochromatographic strip with the enzyme-linked immunosorbent assay (ELISA) kit.

Strip	ELISA Kit			Kappa ^1^	*p*
	Positive	Negative	Total		
Positive	77	6	83		
Negative	4	63	67	0.866	<0.01
Total	81	69	150		

^1^ Kappa is divided into five levels. Kappa = 0.0–0.20, slight agreement; Kappa = 0.21–0.40, fair agreement; Kappa = 0.41–0.60, moderate agreement; Kappa = 0.61–0.80, substantial agreement; Kappa = 0.81–1.00, almost perfect agreement.

**Table 3:** The limit of detection (LOD) and the limit of quantitation (LOQ).

**Mean**	SD	Mean + 2SD	Mean + 10SD	LOD	LOQ
5283.92	355.58	5995.08	8839.70	0.051 ng/mL	0.068 ng/mL

**Table 4:** The results of swine serum.

Serum Name	Result	Judgment	Serum Name	Result	Judgment
PCV2-positive serum	727,198.53	+	PRRSV-positive serum	2351.51	-
PCV2-positive serum	682,285.52	+	PRRSV-positive serum	2556.29	-
PCV2-positive serum	926,893.28	+	PRRSV-positive serum	3165.25	-
PCV2-positive serum	652,288.21	+	PRRSV-positive serum	2885.76	-
PCV2-positive serum	715,582.62	+	PRRSV-positive serum	3515.19	-
PCV2-negative serum	3052.91	-	PEDV-positive serum	2927.51	-
PCV2-negativeserum	3251.82	-	PEDV-positive serum	3992.31	-
PCV2-negative serum	2752.51	-	PEDV-positive serum	3812.15	-
PCV2-negative serum	3192.35	-	PEDV-positive serum	3165.23	-
PCV2-negative serum	2951.59	-	PEDV-positive serum	3079.25	-
PRV-positive serum	3515.36	-	CSFV-positive serum	5018.27	-
PRV-positive serum	2656.17	-	CSFV-positive serum	3312.82	-
PRV-positive serum	3586.62	-	CSFV-positive serum	2980.65	-
PRV-positive serum	2588.66	-	CSFV-positive serum	3796.61	-
PRV-positive serum	3815.63	-	CSFV-positive serum	3188.56	-

**Table 5:** The results of strains.

Serum Name	Result	Judgment	Serum Name	Result	Judgment
PCV2a/LG	866,125.93	+	PEDV (CV777 strain)	3641.83	-
PCV2a/SZ	796,321.65	+	PRV (HB2000 strain)	2306.58	-
PCV2b/SH	621,432.98	+	CSFV (WH-09 strain)	3023.68	-
PCV2d/JH	468,391.27	+	PRRS (JXA1-R strain)	3790.53	-
PCV2d/WZ	428,625.21	+	PPV (WH-1 strain)	2142.19	-
PK-15 cell culture medium	2816.65	-			

## Data Availability

All data are contained within this manuscript.
